# Investigating Word Length Effects in Chinese Reading

**DOI:** 10.1037/xhp0000589

**Published:** 2018-12

**Authors:** Chuanli Zang, Ying Fu, Xuejun Bai, Guoli Yan, Simon P. Liversedge

**Affiliations:** 1 Academy of Psychology and Behavior, Tianjin Normal University, and School of Psychology, University of Central Lancashire; 2 Academy of Psychology and Behavior, Tianjin Normal University; 3 School of Psychology, University of Central Lancashire

**Keywords:** eye movements, word length, character complexity, Chinese reading

## Abstract

A word’s length in English is fundamental in determining whether readers fixate it, and how long they spend processing it during reading. Chinese is unspaced, and most words are two characters long: Is word length an important cue to eye guidance in Chinese reading? Eye movements were recorded as participants read sentences containing a one-, two-, or three-character word matched for frequency. Results showed that longer words took longer to process (primarily driven by refixations). Furthermore, skips were fewer, incoming saccades longer, and landing positions further to the right of long than short words. Additional analyses of a three-character region (matched stroke number) showed an incremental processing cost when character(s) belonged to different, rather than the same, word. These results demonstrate that word length affects both lexical identification and saccade target selection in Chinese reading.

## Public Significance Statement

During reading we make saccadic eye movements in order to fixate words in the high-acuity foveal region of the retina. For each saccade, the oculomotor control system makes decisions about when a saccade should be initiated, and to where the point of fixation should be targeted. Written Chinese does not have spaces between words, and word length is quite short and less variant relative to English text. The present study provides the first well controlled demonstration of word length effects on eye movement control during natural Chinese reading. This study demonstrates that the oculomotor control system is sensitive to Chinese word length information during reading, and Chinese readers are able to use this information in deciding when and where to move the eyes during reading.

Word length is one of the most important factors influencing eye movement control during reading of alphabetic languages. Long words are fixated for longer and are less likely to be skipped than short words (Liversedge & Findlay, 2000; Rayner, 1998, 2009). Word length also influences the amplitude of first-pass saccades into a word, with initial landing positions centered around the Preferred Viewing Location (PVL; Rayner, 1979) being proportionally closer to the word beginning for long than short words (McConkie, Kerr, Reddix, & Zola, 1988). These findings primarily derive from studies of alphabetic languages like English, where interword spaces define the spatial extent of words and provide a salient visual cue for saccadic targeting. Investigation of word length effects is crucial for the development of models of eye movement control in reading such as E-Z Reader (Reichle, 2011) and SWIFT (Engbert & Kliegl, 2011).

Unlike English, in Chinese there are no explicit visual cues like spaces to separate words. Written Chinese text is formed from strings of equally spaced characters. A single character can be a word itself, or combine with other characters to form multicharacter words. According to the Chinese Lexicon (Chinese Linguistic Data Consortium, 2003), 3% of words are one-character words, 64% are two-character words, 18% are three-character words, and the remainder are four or more character words. Chinese words are short, and variance in word length is reduced relative to English. This raises the question of whether word length in Chinese plays as central a role in eye movement control during reading, as in English.

It has been argued that Chinese readers adopt a constant saccade length strategy during reading (e.g., Li, Liu, & Rayner, 2011; Yan, Kliegl, Richter, Nuthmann, & Shu, 2010; see also Li, Zang, Liversedge, & Pollatsek, 2015), moving the eyes forward at a constant length (with some inherent variability), due to most words in Chinese being two characters long. If so, then the initial landing position distribution on each character of a word should be flat. Such a strategy cannot, however, explain why saccades leaving a 4-character word are longer than those leaving two two-character words, as shown by Wei, Li, and Pollatsek (2013). Wei et al. suggest that Chinese readers might adopt a processing-based strategy such that on each fixation readers estimate the number of characters they are processing efficiently, then direct their eyes to the right of those characters. However, Wei et al. did not control target word frequency, meaning that their effects may be driven by word length, or frequency, or both.

Here, we used carefully controlled stimuli to examine effects of word length on eye movement control during Chinese reading. Specifically, we monitored readers’ eye movements as they read sentences containing a one-, two-, or three-character word with similar frequency and contextual predictability. If word length in Chinese affects lexical identification during reading, as has been demonstrated in English reading, we predicted that longer words would attract more fixations than shorter words, and that the increased numbers of fixations would drive increased reading times for measures aggregating first-pass fixations. To be very clear, word length effect in alphabetic language reading is much smaller on first and single fixations on a word, but reliably emerges on gaze durations due to increased probability of readers’ making refixations on longer words. Furthermore, this effect on fixation durations is mostly driven by words with a length more than six letters, probably due to words with fewer letters being skipped more often (e.g., Kliegl, Grabner, Rolfs, & Engbert, 2004; Rayner, Slattery, Drieghe, & Liversedge, 2011). We therefore expected increased gaze duration alongside increased refixation rates for longer than shorter Chinese words, and this effect would be more pronounced for two-versus three-character words than for one-versus two-character words (for more information please see point 1 in the Appendix). Also, when considering regions of text comprising the same number of characters, but in one condition the region formed a single word, whereas in another condition the characters were constituents of more than a single word, we would expect reduced processing times and more fixations (especially in gaze duration and refixation probability) for the former relative to the latter. Finally, if word length in Chinese affects saccade targeting, the probability of skipping will be lower, and the amplitude of incoming first-pass saccades will be greater for long compared to short words.

## Method

### Participants

Thirty native Chinese speakers (mean age = 24 years, *SD* = 2 years; 25 females) with normal or corrected-to-normal vision from Tianjin Normal University participated.

### Apparatus

Eye movements were recorded via an SR Research EyeLink1000 system. Viewing was binocular and movements of the right eye were recorded. Participants were seated 65 cm from a 19-in. monitor, and one Chinese character subtended approximately 1.0° of visual angle.

### Materials and Design

**Figure 1 fig1:**
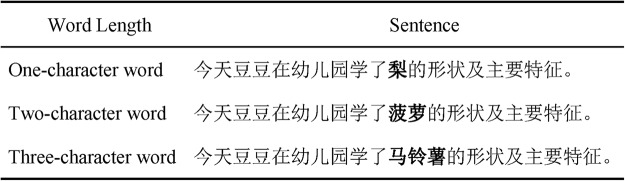
An example of Chinese sentences employed in the experiment (target words are in bold). The translation for this sentence is “Today Doudou learned the **pear/ pineapple/ potato**’s shape and main characteristics in the kindergarten.”

We selected 90 one-, two-, and three-character words from a database developed by Cai and Brysbaert (2010). Frequency of words for each length was closely matched (*M* = 14, 15, and 14, *SD* = 15, 17, and 15 counts per million for one-, two-, and three-character words respectively, *F* = 1).^1^ The number of strokes for one-, two-, and three-character words was 10 (*SD* = 3), 16 (*SD* = 5), and 24 (*SD* = 6) respectively. These differed from each other (*F* = 210), and stroke number was therefore analyzed as a covariate for the target word analyses. We created 90 experimental sentence frames in total, and each sentence frame contained a target word of each length and was identical at least up to the target word (see Figure 1).[Fig-anchor fig1]

Sentences were between 15 and 23 characters long (*M* = 19, *SD* = 2) and were rated for naturalness on a 5-point scale (5 = *very natural*) by 51 participants who did not take part in the eye-tracking study. The mean naturalness score was 4.0 (*SD* = 0.4), with no differences across the three conditions (*F* < 1). Predictability norms from 20 additional participants confirmed that target words of each length were unpredictable from sentence context (*M* = 1%, *SD* = 3%). We constructed three files with each file containing 90 sentences (30 in each condition). Conditions were rotated across files according to a Latin Square. Each participant read experimental sentences presented randomly from one of the three files, with eight practice sentences at the beginning of the experiment. There were 30 yes/no comprehension questions. Based on Westfall (2015), the power of our current design for an average effect size of *d* = 0.45 is 0.861, a value that is greater than the recommended level of 0.8. This suggests our study has good power to establish an effect of average size. We also used a repeated measures experimental design to test more participants with more stimuli per cell than existing studies in the literature that have demonstrated robust word length effects.

### Procedure

Participants read single sentences silently for comprehension, and responded to a yes/no comprehension question occasionally regarding the sentence they had just read. At the beginning of the experiment, a 3-point horizontal calibration procedure was completed, and a drift correction was implemented before the presentation of each sentence and recalibrated as necessary (average calibration error <0.25 degrees). The experiment lasted approximately 20 min.

## Results

Participants’ comprehension accuracy was 94% (*SD* = 4%, with no differences across conditions, all |*z|* < 1). Fixation durations shorter than 80 ms or longer than 1200 ms were deleted from the data set. Trials were removed if there was tracker loss, fewer than three fixations in total (*M* = 12, *SD* = 4, 0.2% of the data), or if measures were above or below 3 SDs from each participant’s mean (1%).^2^

We calculated first fixation duration (FFD, duration of the first fixation on a word), single fixation duration (SFD; fixation duration when only one fixation was made), gaze duration (GD; sum of all first-pass fixations on a word before leaving it), and total fixation duration (TFD; sum of all fixations) as temporal eye movement measures. Spatial measures of eye movements included launch site (position of the previous fixation, measured as the number of characters to the left of the target region), skipping probability (SP), landing positions for single fixations and first of multiple fixations, refixation probability, and incoming saccade length (the length of the saccade entering the target region). Means and standard deviations for the eye movement measures for the target region are shown in Table 1.[Table-anchor tbl1]

We ran linear mixed-effects models (LMMs) using the lme4 package (Version 1.1–12) within the R Environment for Statistical Computing (R Development Core Team, 2014). For all measures, the LMM with the maximum random effects structure (Barr, Levy, Scheepers, & Tily, 2013) was conducted, allowing both random intercepts and random slopes for the word length effect over both participants and items. If the maximum random model did not converge, the model was trimmed by first trimming down the random structure for items, starting with removal of the random effect correlations, then the random slopes. Successive sliding contrasts were carried out, comparing one- with two-character words, and two- with three-character words. Fixation times and saccade length were log-transformed to increase normality of the data. For skipping probability and refixation probability (binary data), logistic GLMMs were carried out. *p* values were calculated based on Satterthwaite’s approximations using the lmerTest package. Fixed effect estimations for the eye movement measures are shown in Table 2.[Table-anchor tbl2]

### Target Word Analyses

**Figure 2 fig2:**
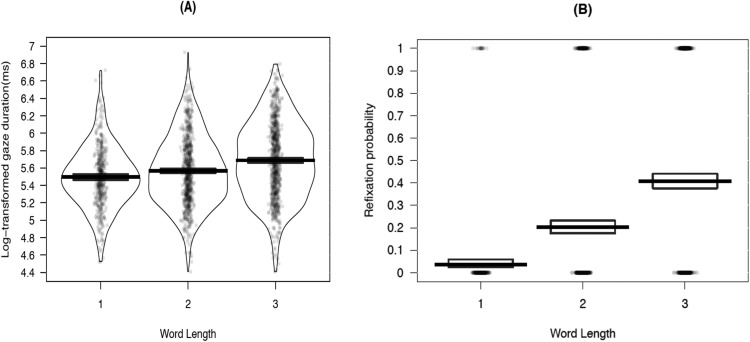
A pirate plot of gaze duration (A) and refixation probability (B) for different word length conditions.

There was no effect of word length on first and single fixation durations on the target word (all |*t*| < 1.41). However, gaze duration^3^ and total fixation duration were significantly longer for three-character words than for two-character words, longer for two-character words than for one-character words on total fixation duration, and numerically longer for two-character words than for one-character words on gaze duration (*t* = 1.07). Furthermore, readers were more likely to make refixations on three-character words than on two-character words, and more on two-character words than on one-character words (all *z* > 5.15). A pirate plot of gaze duration and refixation probability is shown in Figure 2. These patterns suggest that the word length effect was more reliable in gaze and total fixations than first or single fixations because of refixations.[Fig-anchor fig2]

One-character words were more likely to be skipped than two-character words, and two-character words were more likely to be skipped than three-character words (all |*z*| > 5.44). When target words were initially fixated, readers made longer incoming saccades^4^ to three-character words compared to two-character words, and longer saccades to two-character words compared to one-character words (all *t* > 3.13). There were reliable effects on the mean initial landing positions on target words in single fixation cases (all *t* > 9.13) and a marginally reliable difference between two- and three-character words in multiple fixation cases (*t* = 1.92, *p* = .06; for landing position distributions, see also points 3 and 4 in the Appendix). Finally, there was no strong evidence for differences in launch sites for saccades onto the target across the three conditions,^5^ suggesting that landing position effects could not be explained by differences in launch site.

### Processing Cost Analyses

Recall that we predicted that if we were to consider regions of text comprising the same number of characters, but formed from constituent characters from differing numbers of words, we would expect reduced processing times for the single word relative to characters from multiple words. To carry out these analyses, we identified comparable three-character regions of interest: a one-character word followed by two additional characters that may or may not form a word (labeled “1+2 Region,” and comprising on average 2.7 words); a two-character word with an additional character from a different word (labeled “1+1 Region,” and comprising on average 2 words), and a single three-character word (labeled “1 Region”). Stroke complexity was controlled across these regions (*M* = 25, 24, and 24 for “1+2,” “1+1,” and “1” regions respectively, all *p* > .05). The means and standard deviations^6^ for eye movement measures for each of these three-character regions are shown in Table 3, and the corresponding fixed effect estimations are shown in Table 4.[Table-anchor tbl3][Table-anchor tbl4]

The results are straightforward: When an additional character that belonged to a different word was included in the region (1+1 Region), there was additional processing cost relative to a region comprised of a single word (1 Region): 7 ms for first fixation duration, 31 ms for gaze duration, 40 ms for total fixation duration, and 5% for probability of refixation. When two additional characters were included in the region (1+2 Region), the processing cost increased by 10 ms for first fixation duration, 41 ms for gaze duration, 65 ms for total fixation duration, and 5% for probability of refixation. Two aspects of these results are noteworthy. First, the greater the number of characters from different words in a region of comparable size, the greater the cost to processing. This strongly suggests that the appropriate metric of processing cost in Chinese reading is the word, not the character (otherwise, we would expect no difference across the three conditions). This result reinforces the conclusions of Bai, Yan, Liversedge, Zang, and Rayner (2008), who argued that word-based processing is extremely important in Chinese reading. Second, while these differences are robust in terms of processing times, the linear incremental relationship does not hold comparably for the likelihood that readers make refixations. Thus, the effects appear to be driven by decisions about when to terminate fixations rather than decisions to make additional fixations.

## Discussion

Despite Chinese not having spaces between words, and word length being short and less variant relative to English, the length of a word still plays an important role in eye movement control during Chinese reading. Specifically, when only the target word was analyzed (with its stroke number as a covariate), we found that longer words took longer to process, and this effect was mainly driven by more frequent refixations rather than first or single fixation durations (Rayner, 1998, 2009). The absence of a word length effect in first and single fixation duration may be due to the unspaced nature of Chinese text. Boundary information between words in unspaced Chinese text may not be acquired early enough from the parafovea to affect these initial fixations (Li et al., 2011). However, when the same three-character region (with matched stroke number) was analyzed for each word length, there was an incremental processing cost when the additional character(s) belonged to a different word rather than the same word. To reiterate, the greater the number of characters from different words in a region of comparable size, then the greater the processing cost, indicating that processing cost in Chinese reading is most appropriately characterized in relation to word rather than character units (Bai et al., 2008).

The leading models of eye movement control in reading such as E-Z Reader (Reichle, 2011) and SWIFT (Engbert & Kliegl, 2011) do offer an account for word length effects in Chinese reading. Both models implement a visual acuity hypothesis for letter encoding, such that the processing rate of letter recognition decreases linearly with the distance of the letter from the point of fixation. In Chinese, words are comprised of characters, and as word length increases, each constituent character of a word is further away from the high-acuity fovea, and therefore visually degraded. Readers have to make one or more refixations to compensate for this visual acuity limitation and to identify that word efficiently. To this extent, both models can explain the basic findings in the current experiment. However, based on our understanding, neither model can account for the differential processing cost observed for regions of sentences that are the same size (in terms of number of characters), but where those characters derive from different words. If we are correct in this suggestion, then clearly a more nuanced computational algorithm is required to explain these effects.

With respect to saccade targeting, our results are in line with the previous research: Word length is a strong predictor of fixation probability, with long words being skipped less often than short words (Brysbaert, Drieghe, & Vitu, 2005). Word length also affected landing positions in Chinese reading, with fixations landing further into long relative to short words. However, the initial landing position distributions were different from those for reading in alphabetic languages like English. The PVL in English reading is slightly to the left of the center of a word, but the PVL in Chinese shifts from the word center in single-fixation cases to the word beginning in multiple-fixation cases. These patterns are consistent with a series of studies in Chinese reading (see Li et al., 2015). There are several possible explanations for this. The first is that readers may parafoveally identify a word prior to making a saccade to it. If this happens, they may either skip it (especially for single-character words) or target its center based on its length. In contrast, when a parafoveal word is not identified, readers target saccades to the word beginning and then refixate it to continue word identification. A second possible explanation is that readers do not parafoveally identify words, and saccades are targeted to upcoming text in a less informed manner. If a saccade happens to land in an optimal position (close to the PVL), then lexical processing can take place efficiently, and only a single fixation is needed. In contrast, when a saccade lands in a nonoptimal position, word identification is less efficient, and refixations are necessary. The important distinction between these alternative explanations is that in the former, saccadic targeting decisions are made on the basis of lexical information about a parafoveal word, whereas in the latter account, there is no assumption that the parafoveal word has been identified. There is a third possible explanation, however, advocated by Wei et al. (2013). According to this account, saccadic targeting occurs according to a processing-based strategy. On any particular fixation, readers make an estimate of the number of characters that they have efficiently parafoveally processed, and on the basis of this estimate, they target their next saccade to a location just beyond those characters. Wei et al. also showed that when the fixated word is easier to process, then the saccade leaving that word is longest (an effect we replicated here; see Footnote 4). While the current results do not allow us to firmly discriminate between these three theoretical positions, we consider that the latter account may fit most neatly with current and existing data. It is possible that parafoveal processing efficiency judgments are operationalized over visually familiar units, most often, presumably, words (although in principle, larger multiconstituent units of text might be also be sufficiently visually familiar that they may be efficiently processed). If so, then saccadic targeting would occur according to the processing-based account and this would operate most often according to word-based metrics. Note also that the word-based processing accounts fit neatly with our reading time data in this experiment.

In summary, our study provides the first well controlled demonstration of word length effects on eye movement control during natural Chinese reading. When linguistic variables (e.g., word frequency and predictability) and variables related to the visual complexity of text (e.g., the number of strokes in characters; Liversedge et al., 2014; Zang et al., 2016) were carefully controlled, the length of a word was shown to reliably influence both temporal and spatial aspects of eye movement control during Chinese reading, demonstrating that word length affects both lexical identification and saccade target selection, and the effects are observable across a range of alphabetic and logographic systems. Our study also provides further evidence that the oculomotor control system in Chinese reading computes saccade metrics on the basis of words rather than characters, which is compatible with both E-Z Reader and SWIFT. However, explaining our findings of the processing cost might require model changes.

## Figures and Tables

**Table 1 tbl1:** Eye Movement Measures for the Target Word

Word length	FFD	SFD	GD	TFD	ReP	SP	MLPsingle	MLPmultiple	ISL	Launch site
One-character word	247 (83)	249 (84)	261 (103)	321 (175)	.04 (.19)	.48 (.50)	.54 (.28)	.45 (.33)	2.16 (.86)	1.29 (.90)
Two-character word	240 (78)	241 (79)	284 (124)	365 (196)	.20 (.40)	.14 (.35)	.98 (.52)	.53 (.52)	2.36 (.85)	1.42 (.95)
Three-character word	234 (74)	238 (74)	325 (146)	428 (230)	.41 (.49)	.04 (.20)	1.41 (.65)	.74 (.52)	2.47 (.94)	1.38 (.95)
*Note*. Standard deviations are provided in parentheses. FFD = first fixation duration; SFD = single fixation duration; GD = gaze duration; TFD = total fixation duration; ReP = refixation probability; SP = skipping probability; MLPsingle = mean landing position in single fixation cases (characters); MLPmultiple = mean initial landing position in multiple fixation cases (characters); ISL = incoming saccade length (characters).										

**Table 2 tbl2:** LMM Analyses on the Target Word With its Number of Strokes as a Covariate

Contrast	FFD				SFD				GD				TFD				ReP			
	*b*	CI	*SE*	*t*	*b*	CI	*SE*	*t*	*b*	CI	*SE*	*t*	*b*	CI	*SE*	*t*	*b*	CI	*SE*	*z*
One vs. Two	−.02	[−.06, .02]	.02	−.99	−.03	[−.07, .01]	.02	−1.40	.03	[−.03, .09]	.03	1.07	.10	[.03, .17]	.03	**2.91**	1.76	[1.21, 2.31]	.28	**6.28**
Two vs. Three	−.02	[−.05, .02]	.02	−.93	−.03	[−.07, .02]	.02	−1.15	.07	[.02, .13]	.03	**2.63**	.10	[.03, .16]	.04	**2.74**	.78	[.49, 1.08]	.15	**5.16**
Number of strokes	−8e-4	[−.00, .00]	.00	−.57	3e-4	[−.00, .00]	.00	.17	6e-3	[.00, .01]	.00	**3.15**	7e-3	[.00, .01]	.00	**2.69**	6e-2	[.04, .08]	.01	**5.04**
	SP				MLPsingle				MLPmultiple				ISL				Launch site			
Contrast	*b*	CI	*SE*	*z*	*b*	CI	*SE*	*t*	*b*	CI	*SE*	*t*	*b*	CI	*SE*	*t*	*b*	CI	*SE*	*t*
One vs. Two	−1.75	[−2.05, −1.45]	.15	**−11.41**	.44	[.36, .51]	.04	**11.07**	.15	[−.11, .40]	.13	1.14	.10	[.05, .14]	.02	**4.36**	.10	[−.01, .21]	.06	1.74
Two vs. Three	−1.24	[−1.68, −.79]	.23	**−5.45**	.42	[.33, .51]	.05	**9.12**	.15	[−.00, .30]	.08	1.92	.06	[.02, .10]	.02	**3.14**	−.01	[−.13, .11]	.06	−.14
Number of strokes	−3e-2	[−.06, −.00]	.01	**−2.03**	−1e-3	[−.01, .00]	.00	−.37	8e-5	[−.01, .01]	.00	.02	−5e-3	[−.01, −.00]	.00	**−3.03**	−2e-3	[−.01, .01]	.00	−.39
*Note*. Significant items are presented in bold, and marginal significant items are underlined. FFD = first fixation duration; SFD = single fixation duration; GD = gaze duration; TFD = total fixation duration; ReP = refixation probability; SP = skipping probability; MLPsingle = mean landing position in single fixation cases (characters); MLPmultiple = mean initial landing position in multiple fixation cases (characters); ISL = incoming saccade length (characters); LMM = linear mixed-effects models; *b* = regression coefficient; CI = confidence interval.																				

**Table 3 tbl3:** Eye Movement Measures for the Three-Character Region in the Analyses of Processing Cost

Word length	FFD	SFD	GD	TFD	ReP
1+2 Region	251 (85)	253 (88)	405 (215)	549 (306)	.51 (.50)
1+1 Region	241 (77)	245 (75)	364 (178)	484 (271)	.46 (.50)
1 Region	234 (75)	238 (74)	333 (159)	444 (252)	.41 (.49)
*Note*. Standard deviations are provided in parentheses. FFD = first fixation duration; SFD = single fixation duration; GD = gaze duration; TFD = total fixation duration; ReP = refixation probability. 1+2 Region = a one-character word followed by two additional characters that may or may not form a word; 1+1 Region = a two-character word with an additional character from a different word; 1 Region = a single three-character word.					

**Table 4 tbl4:** LMM Analyses on the Three-Character Region in the Analyses of Processing Cost

Contrast	FFD				SFD				GD				TFD				ReP			
	*b*	CI	*SE*	*t*	*b*	CI	*SE*	*t*	*b*	CI	*SE*	*t*	*b*	CI	*SE*	*t*	*b*	CI	*SE*	*z*
“1+2” vs. “1+1”	−.03	[−.06, −.01]	.01	**−2.40**	−.03	[−.07, .02]	.02	−1.17	−.09	[−.14, −.04]	.03	**−3.47**	−.12	[−.18, −.06]	.03	**−4.10**	−.21	[−.43, .01]	.11	−1.89
“1+1” vs. “1”	−.03	[−.06, .00]	.01	−1.92	−.03	[−.07, .00]	.02	−1.74	−.08	[−.13, −.04]	.02	**−3.66**	−.10	[−.16, −.03]	.03	**−3.09**	−.29	[−.50, −.08]	.11	**−2.66**
*Note*. Significant items are presented in bold, and marginal significant items are underlined. FFD = first fixation duration; SFD = single fixation duration; GD = gaze duration; TFD = total fixation duration; ReP = refixation probability; LMM = linear mixed-effects models; *b* = regression coefficient; CI = confidence interval.																				

**Table A1 tbl5:** Word Length Effect in Reading of Alphabetic Scripts and Chinese

Study	Measures	Word length (Number of letters)												Word length effect
		2	3	4	5	6	7	8	9	10	11	12	13	
Kliegl et al. (2004) (estimated from Figs.1 & 2)	Gaze duration	230	230	230	235	230	240	250	270	300	320	340	373	Effect was more pronounced for words with > 6–8 letters
	RefixP	.04	.06	.08	.10	.12	.16	.20	.30	.38	.46	.50	.64	>6 letters
	SkipP	.64	.48	.32	.22	.18	.12	.10	.02	.00	.02	.02	.06	<8 letters
McDonald (2006)	Gaze duration					260		290						30 ms
	RefixP					.20		.25						.05
	SkipP					.12		.09						ns
Hautala et al. (2011)	Gaze duration			208		219								11 ms
	RefixP			.08		.09								ns
	SkipP			.35		.09								.26
Rayner et al. (2011)				Short (4–6 letters)			Medium (7–9 letters)			Long (10–12 letters)				
	Gaze duration			217			222			234				Difference appears between Medium and Long words
	SkipP			.32			.20			.14				all *p* < .05
	Chinese													
		1-character word		2-character word			3-character word			4-character word			2 2-character words	Word length effect
Li et al. (2011)	Gaze duration			266						355				89 ms
	SkipP			.34						.07				.27
Wei et al. (2013)	Gaze duration									502			585	83 ms
	RefixP									.69			.77	.08
*Note.* ns = not significant; RefixP = refixation probability; SkipP = skipping probability.														

**Table A2 tbl6:** Fixation Times and Saccade Length for the Target Word (Log-Transformed Data)

Word length	FFD	SFD	GD	TFD	ISL	OSL
One-character word	5.47 (.34)	5.48 (.34)	5.51 (.37)	5.66 (.50)	.72 (.37)	.62 (.43)
Two-character word	5.44 (.32)	5.45 (.32)	5.58 (.42)	5.78 (.51)	.80 (.36)	.79 (.39)
Three-character word	5.41 (.32)	5.43 (.32)	5.71 (.46)	5.94 (.55)	.85 (.37)	.85 (.37)
*Note*. Standard deviations are provided in parentheses. FFD = first fixation duration; SFD = single fixation duration; GD = gaze duration; TFD = total fixation duration; ISL = incoming saccade length (characters); OSL = outgoing saccade length (characters).						

**Table A3 tbl7:** LMM Analyses on the Target Word With its Number of Strokes as a Covariate (Log-Transformed Data)

Contrast	FFD				SFD				GD			
	*b*	CI	*SE*	*t*	*b*	CI	*SE*	*t*	*b*	CI	*SE*	*t*
One vs. Two	−.03	[−.07, .01]	.02	−1.39	−.04	[−.08, .01]	.02	−1.65	.04	[−.02, .10]	.03	1.37
Two vs. Three	−.03	[−.06, .01]	.02	−1.36	−.04	[−.08, .00]	.02	**−1.85**	.09	[.03, .15]	.03	**2.93**
Number of strokes	−8e-5	[−.00, .00]	.00	−.06	1e-3	[−.00, .00]	.00	.67	6e-3	[.00, .01]	.00	**2.66**
	TFD				ISL				OSL			
Contrast	*b*	CI	*SE*	*t*	*b*	CI	*SE*	*t*	*b*	CI	*SE*	*t*
One vs. Two	.09	[.02, .16]	.04	**2.64**	.09	[.05, .13]	.02	**4.26**	.17	[.11, .23]	.03	**5.54**
Two vs. Three	.11	[.03, .18]	.04	**2.81**	.07	[.03, .11]	.02	**3.73**	.08	[.03, .12]	.02	**3.48**
Number of strokes	8e-3	[.00, .01]	.00	**2.80**	−5e-3	[−.01, −.00]	.00	**−3.48**	−4e-3	[−.01, −.00]	.00	**−2.54**
*Note.* Significant items are presented in bold, and marginal significant items are underlined. FFD = first fixation duration; SFD = single fixation duration; GD = gaze duration; TFD = total fixation duration; ISL = incoming saccade length (characters); OSL = outgoing saccade length (characters); LMM = linear mixed-effects models; *b* = regression coefficient; CI = confidence interval.												

**Figure A1 fig3:**
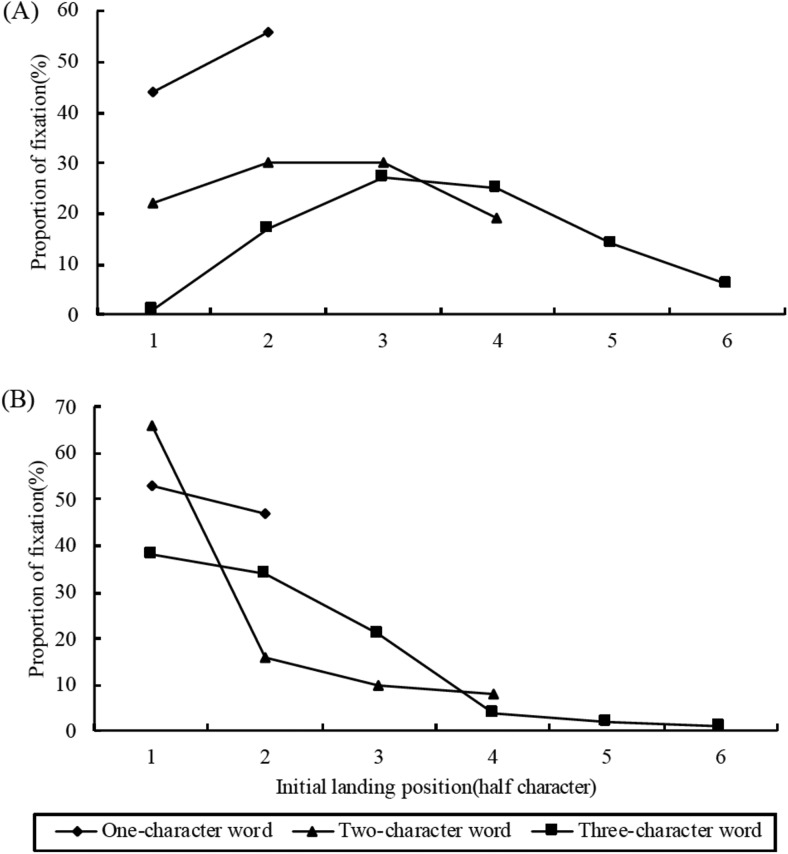
The distribution of landing positions in single fixation cases (A) and in first of multiple fixation cases (B) for different word length conditions. Note, half of a character in the horizontal direction was defined as a unit.

**Figure A2 fig4:**
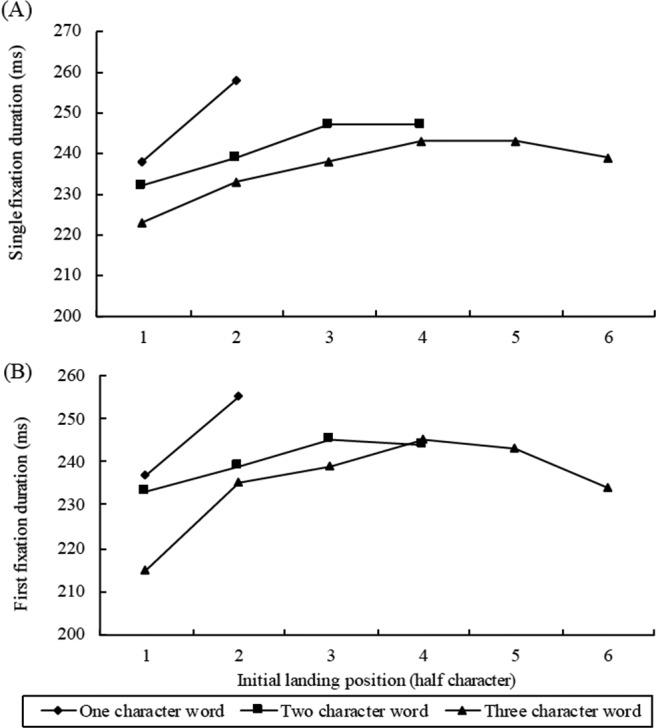
The mean durations of single (A) and first fixations (B) as a function of the initial landing position for different word length conditions.

## Footnotes

1 The first character frequency for one-, two-, and three-character words was 54, 462, and 912 counts per million, *F* = 9.24, *p* < .05. To ensure that the difference in the first character frequency did not explain the findings for the target word, we recomputed the analyses including first character frequency as a co-variate in the LMM (the character frequency was log transformed and centered about its mean due to the nature of the continuous variable). The pattern of results was highly similar to the original analyses. Furthermore, likelihood-ratio tests indicated that, for all measures, the LMM model with first character frequency as a co-variate did not explain significantly more variance than the LMM without it, all χ^2^ < 2.79, *p*s > .05, providing clear evidence for word-based processing during Chinese reading.

2 We reanalyzed our data removing outliers based on the log transformed data, and the pattern of results was almost identical to that reported (for more information please see point 2 in the Appendix).

3 In order to quantify the magnitude of the word length effect in Chinese, we computed how much additional time per character was required to process a word. 95% confidence intervals (CIs) were computed. Processing a one-character word required 261 ms [CI: 242 ms, 274 ms], a two-character word required an additional 23 ms [CI: 10 ms, 38 ms], and a three-character word required an additional 41 ms [CI: 28 ms, 54 ms] compared to a two-character word. These measures provide baseline metrics of the word length effect in Chinese reading.

4 We also analyzed the outgoing saccade length (the length of the saccade leaving the target region) and found that this was longer for three-character words (*M* = 2.45) than for two-character words (*M* = 2.32), and longer for two-character words than for one-character words (*M* = 2.01, all *t* > 2.84). While these results might indicate that a word’s length influenced the execution of a saccade away from it, we note that there were content differences after the target word across our experimental conditions, and therefore any such conclusions should be made cautiously.

5 A Bayesian linear mixed model was fit using the rstanarm package (Stan Development Team, 2016; see Nicenboim & Vasishth, 2016 for an introduction to the method). The prior distribution on the intercept was Normal (0, 15), and the prior distribution on the slopes was Normal (0, 1). Sampling from the posterior distribution was done with 5 Markov Chain Monte Carlo chains with 10,000 iterations each. The first 1,000 iterations were discarded as burn-in. Visual inspection of the traceplots and Gelman and Rubin’s (1992) convergence diagnostic suggested that all models had converged. Bayes factors were calculated using the Savage–Dickey density ratio method described in Wagenmakers, Lodewyckx, Kuriyal, and Grasman (2010) and Nicenboim and Vasishth (2016). Bayes factors greater than 1 favor the null hypothesis, while Bayes factors smaller than 1 favor the alternative hypothesis (3.65 for one- vs. two-character words, 6.94 for two- vs. three-character words). A sensitivity analysis using a range of realistic priors indicated that the choice of prior did not influence the conclusions from this analysis. These results indicate clearly that landing position effects did not arise due to launch site differences.

6 There were subtle differences in terms of the means for three-character words in Table 3 compared with those in Table 1, due to the outliers regarding the three SD criteria being removed separately for the two sets of analyses.

## Additional Literature and Analyses

1 In Table A1, we note word length effects that have been reported in the previous literature that are particularly relevant to our study in relation to alphabetic scripts (Hautala, Hyönä, & Aro, 2011; Kliegl et al., 2004; McDonald, 2006; Rayner et al., 2011) and Chinese (Li et al., 2011; Wei et al., 2013). Unsurprisingly, it is apparent from these studies that the word length effect in alphabetic scripts for gaze duration is mostly driven by words with a length more than six letters. In relation to the Chinese data, the pattern is much less clear due to the very small number of studies that have been carried out that manipulate word length. What can be seen is that there appears to be a complete absence of data relating to effects associated with one- and two-character words, and some suggestion that there are pronounced effects between words of length 2 and 3 or 4 characters. Based on this assessment of the literature, and our understanding of word length effects generally, it was unclear to us why, in principle, length effects could not occur for one- and two-character words in Chinese. This was at least part of the motivation for the present study.[Table-anchor tbl5]

Importantly, it should be noted that we did not adopt some form of equivalence assumption between the orthographic form of alphabetic languages and that of Chinese (e.g., a character = a phoneme, or a character = a morpheme, or some other alternative). We have encountered this form of argument several times before, most notably in Liversedge et al. (2016), where we compared eye movement behavior during reading across three very different languages (English, Finnish and Chinese). After very extensive consideration, we concluded that the adoption of equivalence assumptions was fundamentally flawed (and indeed, our findings demonstrated that past studies that have adopted some form of equivalence algorithm have led to invalid theoretical conclusions). Liversedge et al. refer to this point as the “apples and pears” issue and argue that it is simply inappropriate to form an equivalence metric across the languages because they are so very different in so many respects. To very briefly illustrate our point, consider how we might try to develop such a metric between Chinese and alphabetic languages. First, which alphabetic language are we considering? Two examples might be English and Finnish. Finnish is agglutinative and has a much longer average word length than English, yet many corresponding words in English and Finnish that have quite different word lengths have the same (usually two-character) word in Chinese. Furthermore, there are many single words in Finnish that correspond to multiple words in English and Chinese, and those words are not necessarily spatially adjacent in Chinese. Also, there are multiple instances of orthographic units that exist as words in one language but do not exist as words in others. Furthermore, if we seek equivalence in relation to the phonological form of the languages, we again become unstuck. The phonological characteristics of English and Finnish are very different, and these themselves are quite different to the phonological form of Chinese. Again, forming correspondences between phonological form and word length is potentially misleading.

To reiterate, these are arguments that we have engaged with and directly addressed in the Liversedge et al. paper. It is for these reasons that we did not adopt the equivalence approach when we generated our predictions for the present experiment. We did, however, try to be principled and scientific in our approach, adopting well accepted effect size estimation techniques, and engaging with the empirical question as to whether word length effects might occur in Chinese (particularly in relation to one- and two-character words). In our view, the current experiment provides benchmark empirical data in relation to word length effects in natural Chinese reading.

2 Fixation times and saccade length were reanalyzed removing outliers based on the log-transformed data. The pattern of results was almost identical to that reported; see Tables A2 and A3.
3 Initial landing positions were different in single fixation and multiple fixation cases. The interaction between Word Length (one vs. two-character words) and Fixation Type (single vs. multiple fixation cases) was marginal, *b* = 0.25, *t* = 1.84, *p* = .07, and the interaction between Word Length (two vs. three-character words) and Fixation Type (single vs. multiple fixation cases) was reliable, *b* = 0.17, *t* = 3.00, *p* < .001. The landing position distributions in the two cases are shown in Figure A1. Broadly, readers tended to target saccades toward a word center when only a single fixation was made on that word, but the pattern shifted to saccades targeted to a word beginning when multiple fixations were made on that word. These patterns were consistent with those from previous research (see Li et al., 2015; Zang, Liversedge, Bai, & Yan, 2011 for reviews).[Fig-anchor fig3]
4 The mean durations of single and first fixations as a function of the initial landing position on target words with different lengths are shown in Figure A2. The results show that the position of initial fixations (including single and first of multiple fixations) on a word influences the duration of that fixation, with initial fixations being longest when the eyes are near the center of the word, replicating the Inverted Optimal Viewing Position phenomenon (e.g., Nuthmann, Engbert, & Kliegl, 2007; Vitu, McConkie, Kerr, & O’Regan, 2001).[Fig-anchor fig4]

[Table-anchor tbl6][Table-anchor tbl7]

In order to interpret the fixation duration measures as a function of initial landing position in terms of the word length effect, we categorized each initial landing position for each word length when it fell at the beginning, middle, or end of a word. The results showed that the duration of first and single fixations was longer or marginally longer when the fixation initially fell at the middle compared to the beginning of a word (FFD: *t* = 1.76, *p* = .08; SFD: *t* = 2.09, *p* = .04). However, there were no reliable interactions between landing position and word length, showing that initial landing position did not exert different influences on fixation durations for words of different length.
